# Kidney Involvement in Acute Hepatic Porphyrias: Pathophysiology and Diagnostic Implications

**DOI:** 10.3390/diagnostics11122324

**Published:** 2021-12-10

**Authors:** Andrea Ricci, Claudio Carmine Guida, Paola Manzini, Chiara Cuoghi, Paolo Ventura

**Affiliations:** 1 Internal Medicine Unit, Department of Medical and Surgical Science for Children and Adults, Regional Reference Centre for Diagnosing and Management of Porphyrias, University of Modena and Reggio Emilia, Azienda Ospedaliero-Universitaria Policlinico of Modena, Largo del Pozzo 71, 41124 Modena, Italy; andrewrk92@gmail.com (A.R.); chiara.cuoghi@outlook.com (C.C.); 2 Interregional Reference Center for the Prevention, Surveillance, Diagnosis and Treatment of Porphyria, Nephrology and Dialysis Unit, Scientific Institute for Research and Health Care, Viale Cappuccini, San Giovanni Rotondo, 71013 Foggia, Italy; claudiocarmine.guida@tin.it; 3 Transfusion Medicine and Blood Establishment, Regional Reference Centre for Diagnosis and Management of Porphyrias, University Hospital City of Science and Health of Torino, 10126 Torino, Italy; paola.manzini@gmail.com

**Keywords:** porphyria, kidney, nephropathy, givosiran, aminolevulinic acid, porphobilinogen, porphyrins, chronic kidney disease, nitric oxide, kidney transplantation

## Abstract

Porphyrias are a group of rare disorders originating from an enzyme dysfunction in the pathway of heme biosynthesis. Depending on the specific enzyme involved, porphyrias manifest under drastically different clinical pictures. The most dramatic presentation of the four congenital acute hepatic porphyrias (AHPs: acute intermittent porphyria—AIP, ALAD deficiency, hereditary coproporphyria—HCP, and porphyria variegata—VP) consists of potentially life-threatening neurovisceral attacks, for which givosiran, a novel and effective siRNA-based therapeutic, has recently been licensed. Nonetheless, the clinical manifestations of acute porphyrias are multifaceted and do not limit themselves to acute attacks. In particular, porphyria-associated kidney disease (PAKD) is a distinct, long-term degenerating condition with specific pathological and clinical features, for which a satisfactory treatment is not available yet. In PAKD, chronic tubule-interstitial damage has been most commonly reported, though other pathologic features (e.g., chronic fibrous intimal hyperplasia) are consistent findings. Given the relevant role of the kidney in porphyrin metabolism, the mechanisms possibly intervening in causing renal damage in AHPs are different: among others, *δ*-aminolevulinic acid (ALA)-induced oxidative damage on mitochondria, intracellular toxic aggregation of porphyrins in proximal tubular cells, and derangements in the delicate microcirculatory balances of the kidney might be implicated. The presence of a variant of the human peptide transporter 2 (PEPT2), with a greater affinity to its substrates (including ALA), might confer a greater susceptibility to kidney damage in patients with AHPs. Furthermore, a possible effect of givosiran in worsening kidney function has been observed. In sum, the diagnostic workup of AHPs should always include a baseline evaluation of renal function, and periodic monitoring of the progression of kidney disease in patients with AHPs is strongly recommended. This review outlines the role of the kidney in porphyrin metabolism, the available evidence in support of the current etiologic and pathogenetic hypotheses, and the known clinical features of renal involvement in acute hepatic porphyrias.

## 1. Introduction

Porphyrias are a group of rare disorders originating from an enzyme dysfunction in the metabolic pathway of heme biosynthesis [1]. According to the specific enzyme involved, porphyrias manifest under dramatically different clinical pictures [2]: acute hepatic porphyrias (AHPs: acute intermittent porphyria—AIP, aminolevulinic acid (ALA) dehydratase deficiency porphyria—ALADp, hereditary coproporphyria—HCP, and variegate porphyria—VP) present with potentially life-threatening acute neurovisceral attacks (or acute porphyric attacks—APAs), whereas nonacute porphyrias (porphyria cutanea tarda—PCT and erythropoietic protoporphyria—EPP, among others) mainly display a range of debilitating dermatologic manifestations and—for EPP—a higher risk of developing a chronic hepatic disease (among acute porphyrias, HCP and VP may also present with cutaneous symptoms [3,4]).

Each of the porphyrias is characterized by a specific pattern of accumulation of heme precursors (*δ*-aminolevulinic acid, porphobilinogen—PBG, or porphyrins) in plasma, urine, and/or feces, depending on the hydrophobicity of the different compounds. Since porphyrins are highly reactive to ultraviolet rays, when found in urine they cause it to turn to a reddish hue under sunlight exposure. At the same time, when porphyrins deposit in the skin, they are responsible for the painful phototoxic reactions of cutaneous porphyrias, mainly due to the light-dependent release of cytotoxic reactive oxygen species (ROS) in the course of type I/II photosensitized reactions [5,6].

Due to their heterogeneous and often not specific presentation, added to their utmost rarity, porphyrias represent a notoriously difficult diagnostic challenge for the clinician [7]. Nonetheless, clinical awareness of this group of diseases among physicians is paramount, since patients with porphyrias are heavily burdened not only by their condition, but also by diagnostic delays in the range of months to years, with all the subsequent risks of mistreatment or suboptimal management.

Until recently, treatment options for AHPs were limited to avoiding those environmental stimuli (e.g., fasting, alcohol, “porphyrinogenic” drugs) which, by putatively increasing the metabolic demand for heme, could trigger an APA. In the absence of randomized controlled trials or a shared consensus, periodic infusions of heme arginate have been implemented as a prophylactic therapy for APAs [8], whereas acute attacks are currently managed with heme arginate, glucose infusions, and supportive therapy [8]. It should be underlined that liver transplantation is currently deemed the only curative option for patients with AHPs [9].

In recent years, a novel siRNA-based drug, givosiran, has been approved for the treatment of acute hepatic porphyrias [10,11]: by specifically inhibiting the liver isoform of ALA synthase (ALAS1), the first and rate-limiting enzyme of the heme biosynthetic pathway, givosiran has shown to be highly efficacious in reducing the frequency of porphyric attacks and improving the quality of life of patients with AHPs [12].

While givosiran has represented a breakthrough in the management of acute porphyrias, it must not be overlooked that, other than acute attacks, patients with AHPs develop long-term complications such as chronic neuropathy, hepatocellular carcinoma, and chronic kidney disease (CKD) [13,14]. In fact, porphyria-associated kidney disease (PAKD) has been recognized as a distinct entity with specific pathological and clinical features [15], for instance, most patients with AIP suffer a progressive impairment of kidney function, with an estimated decline in glomerular filtration rate (eGFR) of 1 mL/min per 1.73 m^2^ per year [16].

The purpose of this review is to outline the known features of renal involvement in acute hepatic porphyrias: most of the knowledge on this subject is provided by studies on the most common AHP, acute intermittent porphyria, even though at least a few early observations on the South African cluster of variegate porphyria are available [17,18].

## 2. Role of the Kidney in Porphyrin Metabolism

It is generally assumed that the kidney contributes to heme production as the third major synthesizing organ, after the bone marrow and the liver—which account, respectively, for 80% and 15% of total heme biosynthesis [19]. In fact, several biochemical, ultrastructural, and fluorescence microscopy studies have suggested that the kidney is overall abundant in heme. At variance with the liver, though, the capacity for heme biosynthesis in the kidney is heterogeneously distributed and highly compartmentalized, and parallels the activity of detoxifying cytochromes and other heme-dependent functions [20]. Thus, it has been demonstrated that heme biosynthetic activity and porphyrin concentration in the kidney follow a corticomedullary gradient: both are highest in the cortical proximal tubules, a metabolically active region particularly exposed to xenobiotics or other endogenously produced compounds [20].

Compared to liver cells, ALAS in the kidney is somewhat more refractory to induction by porphyrinogenic stimuli. The induction *process*—an initial increase in the enzyme’s activity in the cytosol and a subsequent shift into the mitochondrial matrix- seems qualitatively similar to what has been observed in the liver, but its *kinetics* are much slower (i.e., hours instead of minutes) [20]. By contrast, renal ALAS activity is promptly inhibited by heme, similarly to the liver isoform. Finally, a greater ratio of ferrochelatase-to-ALAS activity has been detected in renal compared to liver cells. Together with other pieces of evidence, these observations have led to the hypothesis that the kidney could benefit from a higher content of intracellular, regulatory “free” heme, which could also function as a protective buffer to acute heme-depleting stimuli [20].

This being considered, it might be of interest to estimate the amount of the “free” heme pool reserves in liver cells. Liver transplantation is deemed curative in AHPs [9]: therefore, it may be conjectured that the damage in AHP might derive, at least partially, from the tissue-specific lower concentration of intracellular unbound heme in the liver and the subsequent greater susceptibility to induction of hepatic ALAS [20].

Several observations have supported the idea that porphyrin excess in urine, e.g., during attacks, is of renal origin [17,20,21,22,23]. In particular, studies on the kidney’s porphyrin clearance, as well as observations on lead intoxication [24] and on patients with variegate porphyria [24,25], point to a renal endogenous production of coproporphyrin.

## 3. Etiology of Chronic Kidney Disease in Acute Porphyria

Several factors may contribute to the decline in kidney function in patients with AHPs.

In the first instance, it should be considered that hypertension is a common finding in patients with acute porphyrias [13,26,27], and that hypertensive damage, even in the form of repeated hypertensive crises along the course of porphyric attacks, may contribute to the overall decline in renal function in this group [27,28,29]. Nevertheless, patients with AHPs develop kidney impairment even before the onset of hypertension [30].

An observational study on a few hundred patients with *HMBS* mutation (either AIP patients or asymptomatic carriers) confirmed a significant association between the diagnosis of acute intermittent porphyria and chronic kidney disease (CKD) independent of hypertension [16]. In particular, overt AIP was associated with a greater decline in eGFR over time than the asymptomatic carrier state [16]. Compared to asymptomatic carriers, higher levels of urinary neutrophil gelatinase-associated lipocalin, a marker of tubular damage, have been detected in patients with AIP during attacks [16]. Quite interestingly, the authors of this study also pointed out a noticeable association between some specific *HMBS* mutations (e.g., c.291delG) and a greater eGFR decline (compared to, for example, the c.517C > T missense mutation) [16].

A significant impact on the severity and evolution of CKD in individuals with *HMBS* mutation, even if asymptomatic, has been demonstrated for a specific variant of the peptide transporter 2 (PEPT2). PEPT2 is expressed on the brush border of the tubular proximal cells in the S2 and S3 segments, where it acts as a proton-coupled symporter for the reabsorption of di- and tri-peptides, plus a number of peptide-like endogenous and exogenous compounds (e.g., carnitine, antiviral nucleoside prodrugs, cephalosporins), including the highly hydrophilic ALA. The PEPT2*1 variant has a greater affinity to its substrates compared to PEPT2*2, and its presence has been independently associated in multivariate analysis with a worse decline in renal function and lower eGFR in patients with *HMBS* mutations, regardless of the presence of other symptoms of the disease. [31] Since PEPT2 is also expressed on the apical side of the choroidal plexus [32], where it drains ALA from the central nervous system, these results are mirrored by the findings of a neuroprotective function of the PEPT2*1 variant in subjects exposed to a risk of lead toxicity (by inhibiting ALA dehydrogenase, the second enzyme in the pathway of heme biosynthesis, lead recapitulates a form of acquired acute porphyria) [33,34]. Notably, PEPT2 is inhibited by losartan [35], and the use of this angiotensin II receptor blocker has been proposed as a treatment to slow down the progression of porphyria associated kidney disease [31].

## 4. Pathogenesis of Kidney Damage in PAKD

Among several mechanisms by which ALA is thought to cause cytotoxic damage, the kidney may be particularly susceptible—at least in its most metabolically active segments—to mitochondrial ALA-induced oxidation. At the intracellular level, ALA undergoes a phosphate-catalyzed auto-enolization, and becomes an oxidizing agent; it reacts with iron and O_2_ to produce superoxide anion (O_2_), HO radical, and ALA radical (ALA); ALA, in the presence of oxygen, reduces iron and yields dioxo valeric acid (DOVA), a highly reactive oxidant [36,37]. Several pieces of evidence have been gathered concerning ALA toxicity on mitochondrial morphology, loss of transmembrane potential, and protein expression [38,39,40].

Renal histopathological findings in patients with PAKD point toward chronic tubulointerstitial damage [16,18,30,41,42,43] and chronic fibrous intimal hyperplasia associated with focal cortical atrophy [16]. Early autopsy reports in a South African series of patients with variegate porphyria evidenced renal tubular degeneration, more marked in distal tubules, with calcified casts [18]. More recently, Pallet et al. [16] described tubular atrophy, basal membrane thickening, and interstitial fibrosis; nonspecific arteriosclerotic lesions [16] have also been observed, with arterial fibrous intimal hyperplasia in the cortex, consisting of myofibroblast growth, sclero-fibrotic tissue production and endothelial lumen narrowing. Remarkably, glomeruli seem spared from direct damage [43], since only unspecific sclerotic and ischemic lesions have been reported [16,30]. Markers of ongoing fibrogenesis, such as cytoplasmic accumulation of ß-catenin and vimentin expression, [16] have been detected in tubular sections, and mitochondrial abnormalities have been reported anecdotally [18,42].

Cell culture studies have shown that human endothelial cells (HUVECs), when incubated with ALA and PBG, do not appear to suffer direct damage from the porphyrin precursors [16]. In contrast, human renal epithelial cells (HRECs) display a wide range of alterations in the presence of ALA and PBG in vitro, i.e.: activation of apoptosis, with signs of autophagy and endoplasmic reticulum stress; evidence of a proinflammatory and fibrogenic secretory milieu; morphologic and molecular changes suggestive of epithelial-to-mesenchymal transition (loss of the cuboid morphology, cell-to-cell contact, E-cadherin expression; nuclear translocation of β-catenin; increased expression of SLUG).

On electron microscopy, HRECs incubated with PBG showed accumulation of electron-dense cytosolic granules, whereas light microscopy detected yellow-brown granular aggregates, negative for Perl’s stain, and numerous cytoplasmic osmiophilic granules within the proximal tubular cells [16]. Intriguingly, when proximal tubular cells are incubated with PBG, the latter is completely metabolized into uroporphyrinogen I and III [16]: therefore, it has been conjectured that the observed intracellular inclusions could be aggregates of uroporphyrin obtained by the uncatalyzed polymerization and cyclisation of four PBG molecules.

It is then interesting, from a historical as well as a scientific perspective, that a few studies on acute porphyrias from the past century have reported histopathological findings suggestive of tubular deposition of porphyrins [18,44,45,46]; for instance, a case series of autopsies from patients with variegate porphyria mentioned the presence of a brown autofluorescent pigment, not staining as iron, in both casts and renal tubular cells, and detected a red-orange autofluorescence in the lumen and epithelial cells of Henle’s loop, which in the author’s experience could be possibly attributed to porphyrin deposits [18].

As a matter of fact, consistent pieces of evidence have been gathered concerning the cell-damaging effects of light-independent porphyrin-mediated toxicity [47]: in particular, intracellular, extralysosomal porphyrin accumulation engenders protein aggregation through noncovalent, oxygen-dependent, reversible mechanisms [48,49]. A particular susceptibility has been demonstrated, chiefly in hepatocytes, for intermediate filaments (nuclear laminins and cytoplasmatic keratins) [47,50], proteins in the endoplasmic reticulum (e.g., protein disulfide isomerase and calnexin) [51], proteasome regulatory particles, and key glycolytic enzymes, including glyceraldehyde 3-phosphate dehydrogenase [51]. This process could both trigger and be accelerated by the activity of other oxidizing agents (inflammation, redox reactions) [48], so that porphyrins could precipitate the production of reactive oxygen species (ROS) and intracellular protein aggregation without prior photosensitization. Of note, uroporphyrin I is reduced by the P450 cytochrome’s family and by nicotinamide adenine dinucleotide phosphate (NADPH) in a reaction that yields a superoxide radical (O_2_*^−^*) [52,53]. It may be tempting to speculate that similar mechanisms might take place in the cytochrome-rich renal parenchyma, contributing to the renal toxicity of high concentrations of ALA and PBG.

It must be remarked that when a mouse model of AIP was employed to investigate the effects of repeated phenobarbital-induced acute attacks on renal tissues [54] relatively mild unspecific alterations were undisclosed, even in near-total (that is, 5/6) nephrectomized animals. No granular inclusions or signs of tubule-interstitial damage were evidenced, even though the same authors underscore the differences between the experimental setting and the patients’ condition with years of exposure to abnormal levels of porphyrin precursors [54].

From a clinical perspective, signs of proximal tubulointerstitial insufficiency (i.e., proteinuria, impaired erythropoietin production) and of oxidative damage (increased urinary excretion of lipoperoxides), have been anecdotally signaled in porphyric patients [41,43]. A pattern consistent with sodium losses of tubular origin has been detected in patients with variegate porphyria [17]. A case series reported that, during remission from acute attacks, patients with AHPs displayed signs of tubulointerstitial and hypertensive damage, such as poorly concentrated urines (hyposthenuria), and an impairment of the tubular excretory phase, as disclosed by isotopic renography. In this population, four patients had low serum erythropoietin levels, while all of them (11 with AIP, 1 with VP) had low plasma and erythrocyte vitamin B6 (pyridoxal phosphate, PLP) levels. Interestingly, all patients had significant hyperoxalaemia and hyperoxaluria, and an inverse relationship between plasma oxalic acid and erythrocyte vitamin B6 levels was found in AIP patients [41]. Oxalic acid is a product of glyoxylic acid metabolism, whose conversion to glycine is effected by PLP-dependent transaminases [55,56]. Inherited excessive urinary excretion of oxalic acid (primary hyperoxaluria) is linked to an increased risk of urolithiasis (formation of calcium oxalate kidney stones) and kidney damage [57]. Even though the efficacy of PLP supplementation in reducing oxaluria is debated [56,57,58,59,60], AHPs patients are known to suffer from a poorer vitamin B6 status [61,62] compared to the general population.

## 5. Excretion of Heme Precursors and Kidney Transplantation in End-Stage PAKD

Selectively higher urinary PBG values in association with a decreased kidney function have been observed in patients with *HMBS* mutation [16] and confirmed in a mouse model of AIP [63], where near-total nephrectomy caused the PBG/ALA ratio to increase manifold during porphyric crises, compared to AIP mice with normal kidney function [54]. This tendency is reflected and exacerbated in patients with acute porphyrias and end-stage renal disease (ESRD): in those undergoing hemodialysis, the urinary PBG/ALA ratio increases dramatically between sessions [64], with a subsequent drop after dialysis (dialysis membranes filter both ALA and PBG). Compared to those on hemodialysis, the increase in PBG/ALA ratio has been reported as somewhat less accentuated in a patient undergoing peritoneal dialysis, perhaps as a reflection of a more physiological clearance of the porphyrin precursors [64].

The reason for this selective accumulation of PBG is not entirely clear: it has been supposed that fairly high initial levels of plasma PBG, due to renal impairment, could exert an additional substrate inhibition on hepatic HMBS, precipitating substrate accumulation [54,64]. In fact, HMBS necessitates the assembly of a dipyrromethane cofactor to function properly [65], and excessively high levels of PBG may interfere with this process [66]. In this regard, it is interesting that in the mouse model of AIP, total nephrectomy resulted in a marked decrease in hepatic HMBS activity [54], with maintained transcriptional and protein expression levels.

Probably due to their binding to plasma albumin and hemopexin [67], porphyrins are poorly filtered by dialysis membranes [64,68], even though better performances have been reported with high-flux and high-permeability membranes [69,70]. Patients with acute porphyrias and end-stage renal disease (ESRD) undergoing dialysis show a severe accumulation of porphyrins, almost unaffected by dialysis. These high levels of porphyrins are produced from the nonenzymatic polymerization of four molecules of PBG to hydroxymethylbilane, a linear tetrapyrrole, which spontaneously self-reacts to yield uroporphyrin I, a cyclic tetrapyrrole [68]. In this setting, patients often develop cutaneous signs reminiscent of porphyria cutanea tarda [68], with increased skin fragility and blistering lesions in sun-exposed areas caused by the photo-toxic reaction of the excess of uroporphyrins.

Concerning peritoneal dialysis, it has been recently reported [71] the interesting case of an elderly AIP patient with end-stage CKD and a history of frequent APAs, who experienced a complete discontinuance from attacks after peritoneal dialysis was started and continued on a regular basis—the patient died a few months after the start of dialysis due to the worsening of his severely disabling chronic conditions. The authors of this case report underline that, at variance with most others, this patient’s dialysis fluid did not contain glucose.

Kidney transplantation has been successfully attempted in patients with AHPs and ESRD [68,72,73,74]: most interestingly, transplanted patients experienced an overall clinical improvement in the burden of the disease, with a reduction in the number of porphyric attacks per year, other than the disappearance of the skin lesions [68]. A few cases of combined liver and kidney transplantations have also been reported, with satisfying results (as mentioned before, hepatic transplantation is deemed curative in patients with AHPs) [75,76].

The beneficial effects of kidney transplantation alone are likely due to the interruption of the vicious cycle of accumulation of neuro- and nephrotoxic porphyrin precursors caused by the impairment in renal function. In fact, kidney allograft permitted a more effective clearance of ALA and PBG, which resulted in a reduction in their ratio toward values normally observed in AIP patients with normal kidney function. Moreover, transplanted kidneys are an exogenously implanted source of effective heme synthesis, and could provide some form of compensation for the deficiencies of the heme synthetic pathway at the hepatic level. 

In the setting of organ transplantation, several immunosuppressive drugs have proved safe in patients with porphyria [68,72,73,74]. Of note, trimethoprim/sulfamethoxazole is porphyrinogenic [77] (even if anecdotally used with no consequences [68]), so other agents (e.g., aerosolized pentamidine [73]) should be employed to prevent opportunistic infections.

## 6. Givosiran and PAKD

A novel siRNA-based agent, givosiran, exploits the native RNA-induced silencing complex (RISC) to specifically modulate liver ALAS1 mRNA translation [10,11,12]. Givosiran has shown excellent results in terms of reduction in acute attacks per year and an overall improvement of quality of life in symptomatic patients with AHP [10,11].

At the same time, additional caution should be exercised as to avoid further deterioration in kidney function in patients treated with givosiran [78]. In France, a population of patients with AIP under siRNA-based therapy has been monitored since the beginning of the treatment [79]. Albeit with large fluctuations among subjects, a transient increase in average serum creatinine was reported, with a subsequent stabilization around values slight above the basal. The decrease in renal function occurred usually within three months from the start of givosiran. 

Importantly, none of the patients qualified for a diagnosis of acute kidney injury, based on the KDIGO criteria [80], and those with a pre-existent chronic kidney disease seemed more susceptible to a further deterioration in kidney function. 

Those patients who were followed up for 30 months could be split into the following groups: two patients did not suffer any reduction in the estimated glomerular filtration rate; three patients displayed a modest decline in renal function (−3.4 mL/min/1.73 m^2^/year in average), which could be likely explained by the natural history of PAKD; lastly, two patients experienced an abnormally deep decline in renal function (−5.8 mL/min/1.73 m^2^/year in average). Notably, a kidney biopsy was performed in one of the two patients with progressively declining renal function, yielding no specific signs of active tubular, glomerular, or arteriolar injury, only mild tubular atrophy and interstitial fibrosis were observed; additionally, no immune deposits or alterations of ALAS1 mRNA and protein expression were detected. In this patient, an interruption of treatment with givosiran (due to a pregnancy plan) resulted in a stabilization of renal function.

The authors of this study underscore that givosiran did not alter basal blood pressure; furthermore, no hematuria, leukocyturia, or alterations in average urine protein concentration were recorded. When measured, Urinary Retinol Binding protein, a sensitive marker of proximal tubular injury, was normal.

Different hypotheses are put forward concerning an explanation for the observed effects of givosiran on kidney function [79]. Assuming a misdirected internalization (e.g., by endocytosis) in renal other than liver tissues, givosiran could exert a heme- depleting effect on kidney cytochromes or other hemoproteins such as catalases and peroxidases [81]. Even though pharmacokinetic studies on chronic ALA/PBG “high excreters” disclosed no significant interference of givosiran with the activity of the main hepatic heme-dependent cytochromes of the P450 family [82] (reduction in activity was moderate for CYP1A2 and CYP2D6, weak for CYP2C19 and CYP3A4, and null on CYP2C9), perhaps some of the secondary routes of heme utilization, e.g. in the kidney, might be effectively impaired by the inhibition of ALAS activity and the partial depletion of the intracellular ”free” heme pool.

For the same reasons, an alteration in the vasoactive effects of nitric oxide synthases (NOS), soluble guanylyl cyclase (sGC) or other hemeproteins could impact the delicate microcirculatory balances of the kidney. These alterations could be engendered, if not by relative heme depletion, even by hemodynamic rearrangements because of givosiran interrupting the patients’ chronic exposure to high levels of ALA—a molecule with known vasoactive effects which, at least in the mouse brain, was shown to induce all three NOS isoforms [83].

When heme depletion was induced in animal models by administration of succinyl acetone (another inhibitor of ALA dehydrogenase), the activities of renal nitric oxide synthase [84,85] and soluble guanylate cyclase [85], and the vascular sensitivity to vasodilatory stimuli [85] were significantly reduced, even though no overt alterations in basal blood pressure could be detected, suggesting the presence of multiple compensatory mechanisms.

Additionally, it should be mentioned that patients treated with givosiran have shown increased homocysteine levels, or a further aggravation of their basal hyperhomocysteinemia [86], nonetheless amenable to a therapy of vitamin supplementation [87]. Also in this case, at the moment of writing a status of drug-induced relative heme deficiency cannot be ruled out as a cause for these findings. In fact, cystathionine β-synthase, the first enzyme of the transulfuration pathway (one of the main routes for homocysteine catabolism), relies on heme as a regulatory factor; furthermore, when heme arginate was administered to givosiran-treated patients with severe hyperhomocysteinemia, a transient drop in plasma homocysteine levels was recorded [88]. In theory, by additionally impairing kidney function, a double effect of givosiran on worsening the patients’ homocysteine status should also be considered.

Given the effectiveness of givosiran as a treatment for prevention of APAs, it is highly relevant to precisely characterize the adverse events possibly associated with this therapy. Likewise, because of the intrinsic risk factors of patients with AHPs for developing CKD, postmarketing clinical data are probably necessary before giving a definitive answer for a putative additional decline in kidney function under siRNA-based therapy.

## 7. Conclusions

**Figure 1:**
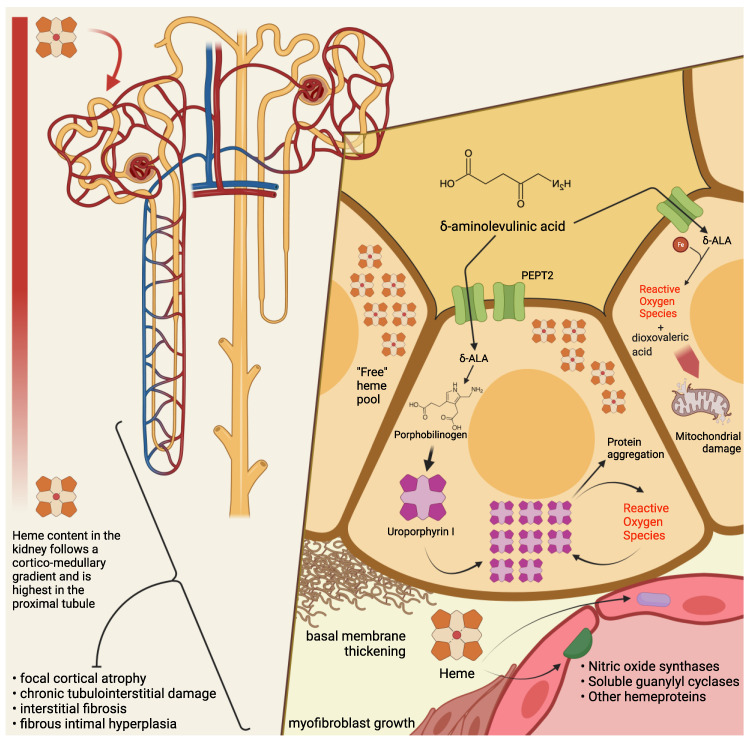
**Mechanisms of kidney damage in acute hepatic porphyrias.** The capacity for heme biosynthesis in the kidney parallels the activity of detoxifying cytochromes and other heme-dependent functions. Compared to the liver, the kidney could benefit from a higher content of intracellular regulatory “free” heme, serving as a protective buffer to acute heme-depleting stimuli. Histopathological findings in patients with porphyria-associated kidney disease have shown chronic tubulointerstitial damage and chronic fibrous intimal hyperplasia associated with focal cortical atrophy. PEPT2 is a proton-coupled symporter expressed on the brush border of tubular proximal cells: the PEPT2*1 variant has a greater affinity to its substrates (including *δ*-aminolevulinic acid—ALA) compared to PEPT2*2, and its presence has been independently associated with a worse decline in renal function in patients with *HMBS* mutation. At the intracellular level, ALA undergoes a multistep reaction in the presence of iron and O_2_ to produce dioxovaleric acid, a highly reactive oxidant: among others, this is a mechanism by which ALA could exert its mitochondrial toxicity. When proximal tubular cells are incubated with porphobilinogen (PBG), the latter is completely metabolized into uroporphyrinogen I and III: thus, the uncatalyzed polymerization and cyclisation of four PBG molecules may lead to the intracellular accumulation of (uro)porphyrin aggregates. Porphyrins have been shown to produce reactive oxygen species (ROS) and cause intracellular protein aggregation without prior photosensitization; this process could both trigger and be accelerated by an oxidizing milieu. Relative heme depletion, as well as hemodynamic rearrangements due to sudden alterations in the levels of circulating ALA, could impact the delicate microcirculatory balances of the kidney regulated by nitric oxide synthases (NOSs), soluble guanylyl cyclases (sGC) or other hemeproteins with vasoactive effects. Other possible mechanisms of kidney damage are discussed in the text. Created with BioRender.com (last accessed date: 5 December 2021).

The clinical manifestations of acute porphyrias are multifaceted and do not limit themselves to acute attacks. In particular, porphyria-associated kidney disease is a long-term, degenerating condition, for which a satisfactory treatment is still not available. A deeper understanding of the mechanisms of kidney damage in AHPs (Figure 1) is crucial for tailoring a treatment aimed at preventing progression to ESRD in these patients.

In fact, a baseline evaluation of kidney function should always be included in the diagnostic workup of AHPs, and we strongly recommend a periodic monitoring of the progression of kidney damage in these patients, whether they are under siRNA-based therapy or not.

Since acute attacks are the most dramatic manifestation of AHPs, a great deal of the research in this field has focused on the pathogenesis and treatment of neurovisceral damage [89]. In this regard, givosiran has truly represented a game-changer in decreasing the rate of attacks and improving the patients’ quality of life. Notwithstanding the concern for some possibly drug-related adverse events, suspension of treatment should be weighed against the heavy burden of the reoccurrence of potentially life-threatening APAs.

In our opinion, the advent of an effective treatment for acute attacks emphasizes the importance of widening our knowledge on the chronic burden of AHPs, e.g., with additional studies on the several available animal models [90]. Furthermore, one focus of research could be how to avoid further worsening of renal function in patients treated with givosiran. In particular, it may be pivotal to gather additional data about the natural history of renal function in large cohorts of patients with AHPs, in order to more precisely compare the effects of siRNA-based therapy in this population.

Finally, it should not be overlooked that acute porphyrias represent a paradigm for the study of heme metabolism and its dysfunctions. Further research in the field of kidney damage related to derangements of heme biosynthesis could prove highly valuable in several contexts and pave the way for wider clinical applications.

## Acronyms

**AHP**	Acute hepatic porphyrias
**AIP**	Acute intermittent porphyria
**ALA**	Aminolevulinic acid
**ALAD**	Aminolevulinic acid dehydratase
**APA**	Acute porphyric attack
**CKD**	Chronic kidney disease
**DOVA**	Dioxovaleric acid
**eGFR**	Estimated Glomerular Filtration Rate
**ESRD**	End-Stage Renal Disease
**HCP**	Hereditary coproporphyria
**HMBS**	Porphobilinogen-deaminase or hydroxymethylbilane-synthase
**HREC**	Human Renal Epithelial Cells
**HUVEC**	Human Umbilical Vein Endothelial Cells
**KDIGO**	Kidney Disease: Improving Global Outcomes
**mRNA**	Messenger RNA
**NADPH**	Nicotinamide adenine dinucleotide phosphate
**NOS**	Nitric oxide synthase
**PAKD**	Porphyria-associated kidney disease
**PBG**	Porphobilinogen
**PEPT2**	Human Peptide Transporter 2
**PLP**	Pyridoxal phosphate
**RISC**	RNA-induced silencing complex
**RNA**	Ribonucleic Acid
**ROS**	Reactive Oxygen Species
**sGC**	Soluble guanylyl cyclase
**siRNA**	Small interfering RNA
**VP**	Variegate porphyria
